# *QuickStats:* Death Rates* from Influenza and Pneumonia^†^ Among Persons Aged ≥65 Years, by Sex and Age Group — National Vital Statistics System, United States, 2018

**DOI:** 10.15585/mmwr.mm6940a5

**Published:** 2020-10-09

**Figure:**
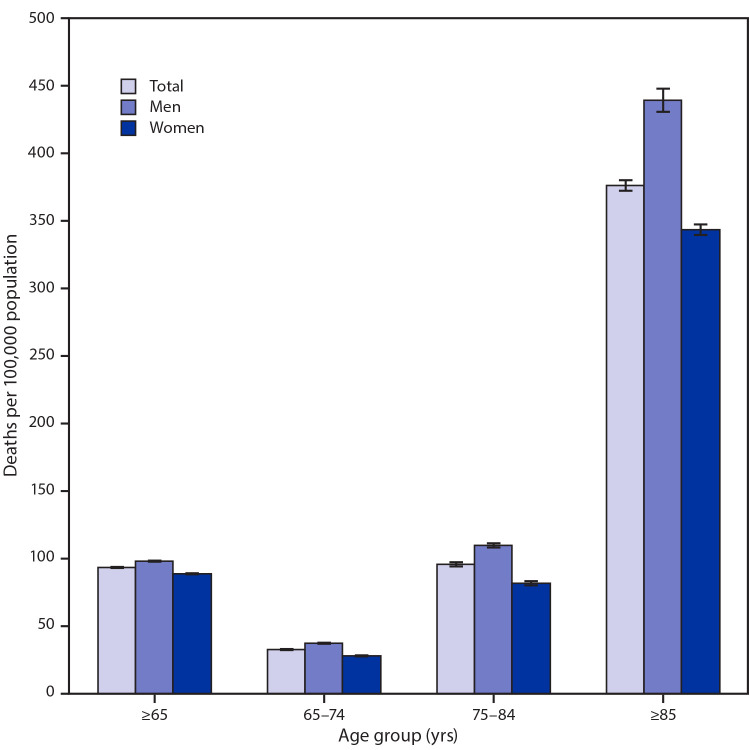
In 2018, the death rate from influenza and pneumonia among persons aged ≥65 years was 93.2 deaths per 100,000 population. Death rates increased with age from 31.7 deaths per 100,000 population among adults aged 65–74 years, to 94.2 among adults aged 75–84 years, to 377.6 among those aged ≥85 years. Rates increased with age for both men and women, and in each age group the death rates were higher for men than for women.

**Source:** National Vital Statistics System mortality data. https://www.cdc.gov/nchs/nvss/deaths.htm.

